# Trends in Utilization of Transitional Care Management in the United States

**DOI:** 10.1001/jamanetworkopen.2019.19571

**Published:** 2020-01-22

**Authors:** Leah M. Marcotte, Ashok Reddy, Lingmei Zhou, Sophie C. Miller, Carly Hudelson, Joshua M. Liao

**Affiliations:** 1 Department of Medicine, University of Washington School of Medicine, Seattle; 2 Value & Systems Science Lab, Seattle, Washington; 3 Leonard Davis Institute of Health Economics, University of Pennsylvania, Philadelphia

## Abstract

This economic evaluation uses Medicare claims data to evaluate changes in utilization of and Medicare payments for transitional care management services from 2013 to 2018.

## Introduction

Improving care transitions after hospitalization is a key opportunity to improve health care value. Recognizing the importance and complexity of coordinating care during the postdischarge period, Medicare implemented transitional care management (TCM) codes in 2013 to increase reimbursement to ambulatory clinicians treating patients after hospital discharge. Early evidence has suggested that TCM could be beneficial, as it was associated with lower costs of care and mortality and readmission rates, although uptake was low.^1,2^ Little is known about national trends in TCM use over an extended period, particularly amid intensifying shifts toward value-based payment and care under federal policies, such as the Medicare Access and CHIP Reauthorization Act of 2015.

## Methods

This economic evaluation used publicly available Medicare claims data from January 1, 2013, to December 31, 2018, capturing 100% of paid and denied TCM services billed to Medicare by physicians nationwide.^3^ For each year, we calculated total service counts and payments for TCM (*Current Procedural Terminology* codes 99495 and 99496), as well as counts and potential payments for denied services. We also compared utilization and payment by physician specialty, classified as primary care physicians (ie, internal medicine, family medicine, general practitioners, and geriatric medicine physicians), medical subspecialists, or other specialty physicians, and by site-of-service, classified as physician office, hospital outpatient department, home, or other. We used χ^2^ tests to compare categorical variables and Kruskal-Wallis tests to compare continuous variables. Statistical tests were 2-tailed and considered significant at an α of .05. Analyses were performed in SAS statistical software version 9.4 (SAS Institute). Per institutional policy at the University of Washington, Seattle, institutional review board approval was not required for this study given the publicly available, deidentified nature of the data. Our analysis followed Consolidated Health Economic Evaluation Reporting Standards (CHEERS) reporting guidelines where applicable.

## Results

Use of TCM increased from 476 307 services provided nationwide in 2013 to 1 358 697 services in 2018, and a total of 5 354 427 TCM service claims were filed during this period. A total of 298 536 TCM services (62.7%) were accepted and $56 476 896 in payments were provided in 2013, which increased to 1 291 827 TCM services (95.1%) accepted and $243 277 363 payments provided in 2018 (Figure). Across this period, 400 864 billed TCM claims (7.5%) were denied by Medicare. In 2013, 177 771 TCM services (37.3%), reflecting $49 705 979 in potential payments, were billed by physicians but denied by Medicare. In 2018, 66 870 TCM services (4.9%), reflecting $20 025 499 in potential payments, were denied (Table).

**Figure.**
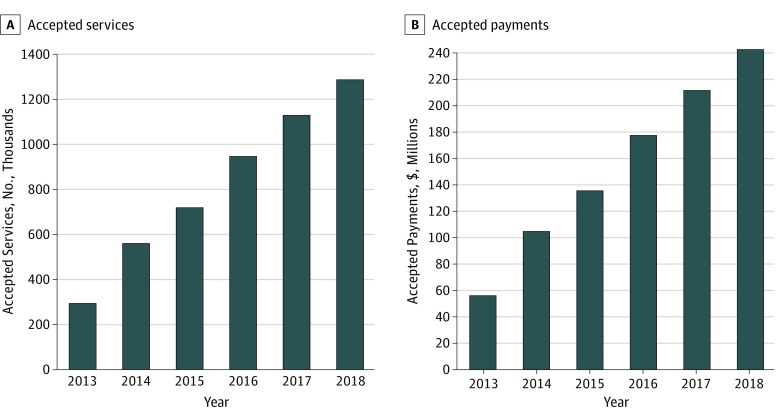
Trends in Transitional Care Management Use and Payment From 2013 to 2018

**Table.** Accepted and Denied Transitional Care Management Services from 2013 to 2018

Year	Total Services, No.	Accepted		Denied	
		Services, No. (%)	Payments, $	Services, No. (%)	Potential Payments, $
2013	476 307	298 536 (62.7)	56 476 896	177 771 (37.3)	49 705 979
2014	593 920	561 182 (94.5)	105 159 993	32 738 (5.5)	10 571 872
2015	763 752	723 074 (94.7)	136 004 897	40 678 (5.3)	13 452 208
2016	988 863	949 420 (96.0)	178 037 644	39 443 (4.0)	13 391 831
2017	1 172 888	1 129 524 (96.3)	212 198 775	43 364 (3.7)	14 898 763
2018	1 358 697	1 291 827 (95.1)	243 277 363	66 870 (4.9)	20 025 499
Total	5 354 427	4 953 563 (92.5)	931 155 568	400 864 (7.5)	122 046 152

Between 2013 and 2018, TCM was most commonly delivered by primary care physicians (4 077 949 services [82.3%]). Among these physicians, utilization increased from 260 899 TCM services (87.4%) in 2013 to 1 024 366 TCM services (79.2%) in 2018, representing an increase from $49 768 824 to $197 181 860 in payments. Overall, 318 994 billed TCM services (7.3%) performed by primary care physicians were denied. With respect to site of service, most TCM services (4 307 821 TCM services [86.9%]) during this period occurred in physician offices. This was also true for each study year, with office-based services composing 271 756 TCM services (91.0%) in 2013 and 1 108 636 TCM services (85.8%) in 2018. Only 114 793 TCM services (2.3%) occurred in a home setting, with a slight increase from 4642 TCM services (1.6%) in 2013 to 31 426 TCM services (2.4%) in 2018 (*P* < .001).

## Discussion

Use of TCM increased between 2013 and 2018 among Medicare beneficiaries, with most services performed by primary care physicians in office settings, while the number of denials by Medicare decreased during this period. Together with the potential scope of TCM—with 5.8 million of 33.7 million Medicare beneficiaries experiencing TCM-covered hospitalizations annually^4^—these results reflect Medicare’s focus on TCM amid efforts to increase reimbursement for care coordination services.^5^ Our findings also highlight potential opportunities to increase specialists’ use of TCM among patients with complex chronic conditions after hospital discharge (eg, cardiologists managing congestive heart failure), as well as to encourage TCM use in other settings (eg, home-based care). This study has limitations, including descriptive design, lack of granular practice- and patient-level data, and inability to evaluate the association of TCM use with patient outcomes. Nonetheless, our results complement recent efforts by Medicare to increase TCM payments^6^ and highlight the need for more research evaluating TCM amid value-based payment and delivery reform.
